# Targeting Polycystic Ovary Syndrome (PCOS) Pathophysiology with Flavonoids: From Adipokine–Cytokine Crosstalk to Insulin Resistance and Reproductive Dysfunctions

**DOI:** 10.3390/ph18101575

**Published:** 2025-10-18

**Authors:** Sulagna Dutta, Pallav Sengupta, Sowmya Rao, Ghada Elsayed Elgarawany, Antony Vincent Samrot, Israel Maldonado Rosas, Shubhadeep Roychoudhury

**Affiliations:** 1 Basic Medical Sciences Department, College of Medicine, Ajman University, Ajman 3464, United Arab Emirates; 2 Department of Biomedical Sciences, College of Medicine, Gulf Medical University, Ajman 4184, United Arab Emirates; 3 School of Life Sciences, Manipal Academy of Higher Education (MAHE), Dubai 345050, United Arab Emirates; 4 Department of Medical Physiology, Faculty of Medicine, Menoufia University, Shebeen Elkom 6131567, Menofia Governorate, Egypt; 5 Department of Microbiology, Faculty of Medicine, Manipal University College Malaysia, Melaka 75150, Malaysia; 6 Citmer Reproductive Medicine, Mexico City 11520, Mexico; 7 Department of Life Science and Bioinformatics, Assam University, Silchar 788011, India

**Keywords:** polycystic ovary, adipokine secretion, cytokine-mediated inflammation, insulin resistance, quercetin, isoflavones, catechins

## Abstract

Polycystic ovary syndrome (PCOS) represents one of the most prevalent endocrine–metabolic disorder in women of reproductive age, which includes but not restricted to reproductive disruptions, insulin resistance (IR), hyperandrogenism, and chronic low-grade inflammation. Its heterogeneous pathophysiology arises from the interplay of metabolic, endocrine, and immune factors, including dysregulated adipokine secretion, cytokine-mediated inflammation, oxidative stress (OS), and mitochondrial dysfunction. Current pharmacological therapies, such as metformin, clomiphene, and oral contraceptives, often provide partial benefits and are limited by side effects, necessitating the exploration of safer, multi-target strategies. Flavonoids, a structurally diverse class of plant-derived polyphenols, have gained attention as promising therapeutic candidates in PCOS due to their antioxidant, anti-inflammatory, insulin-sensitizing, and hormone-modulating properties. Preclinical studies in rodent PCOS models consistently demonstrate improvements in insulin sensitivity, normalization of ovarian morphology, restoration of ovulation, and reduction in hyperandrogenism. Human clinical studies, though limited in scale and heterogeneity, report favorable effects of flavonoids such as quercetin, isoflavones, and catechins on glucose metabolism, adipokine balance, inflammatory markers, and reproductive functions. This evidence-based study critically synthesizes mechanistic insights into how flavonoids modulate insulin signaling, adipokine–cytokine crosstalk, OS, and androgen excess, while highlighting translational evidence and emerging delivery systems aimed at overcoming bioavailability barriers. Collectively, flavonoids represent a promising class of nutraceuticals and adjuncts to conventional therapies, offering an integrative strategy for the management of PCOS.

## 1. Introduction

Polycystic ovary syndrome (PCOS) is constantly soaring as a highly prevalent endocrine–metabolic disorder among women of reproductive age, currently affecting an estimated 8–13% globally, with considerable variation depending on diagnostic criteria and ethnicity [1]. Its heterogeneous presentation, encompassing reproductive dysfunctions such as anovulation and hyperandrogenism, along with metabolic disturbances including obesity, insulin resistance (IR), and dyslipidemia, reflects distinct PCOS phenotypes, each differing in metabolic and inflammatory profiles that complicate diagnosis, management, and long-term care [1,2]. Despite the availability of pharmacological interventions, such as metformin, oral contraceptives, and ovulation-inducing agents, treatment outcomes remain suboptimal, often limited by adverse effects, non-response in subsets of patients, and lack of sustainable long-term benefits [3].

At the core of PCOS lies a multifaceted pathophysiology, where metabolic, endocrine, and immune axes converge [4]. IR and compensatory hyperinsulinemia exacerbate ovarian androgen production, while adipose-derived adipokines and pro-inflammatory cytokines sustain a state of chronic low-grade inflammation [5]. Concomitant oxidative stress (OS) and mitochondrial dysregulations further compromise granulosa cell survival, oocyte competence, and endometrial receptivity [6]. This interconnected network highlights PCOS not as a single-pathway disease but as a systemic disorder with overlapping metabolic, reproductive, and inflammatory dimensions [7].

Given the complexity of PCOS, there is growing interest in identifying therapeutic strategies that can simultaneously modulate multiple pathophysiological pathways [8]. Natural flavonoids, a diverse group of plant-derived polyphenolic compounds, have attracted growing clinical and scientific interest due to their wide-ranging biological activities, including antioxidants, anti-inflammatory, insulin-sensitizing, and hormone-regulating effects, that directly correspond to key pathological processes in PCOS. Their structural diversity, safety profile, and ability to modulate multiple molecular targets make them particularly promising among bioactive phytochemicals for developing integrative therapeutic strategies against PCOS [9]. Unlike conventional single-target agents, flavonoids possess structural diversity and pleiotropic mechanisms of action that allow them to intervene at different nodes of the PCOS network [10].

Despite growing evidence on the therapeutic potential of flavonoids, the mechanistic understanding of how these compounds modulate key PCOS-related pathways, particularly the adipokine–cytokine network, insulin signaling, and ovarian dysfunction, remain fragmented. Furthermore, the limited integration of preclinical and clinical data has hindered the translation of findings into practical interventions. This evidence-based study aims to bridge these gaps by comprehensively summarizing the multi-level actions of flavonoids across metabolic, inflammatory, and reproductive axes of PCOS, linking mechanistic insights with translational perspectives to inform future nutraceutical and therapeutic development.

Relevant literature was identified through a comprehensive search of databases including PubMed, SCOPUS, and Web of Science up to June 2025, using keywords such as “flavonoids”, “polycystic ovary syndrome”, “insulin resistance”, and “inflammation”. Both preclinical (in vitro and animal) and clinical studies evaluating the role of flavonoids in PCOS or related metabolic and reproductive disorders were included. Priority was given to peer-reviewed studies providing mechanistic insights or translational relevance.

## 2. Pathophysiological Basis of Polycystic Ovary Syndrome (PCOS)

### 2.1. Insulin Resistance (IR) and Hyperinsulinemia

IR is one of the major role players in the pathogenesis of PCOS, reported in up to 70% of affected women, irrespective of obesity status [11]. At the molecular level, IR in PCOS is characterized by impaired insulin receptor substrate-1 (IRS-1) phosphorylation and dysregulated phosphatidylinositol 3-kinase (PI3K)/protein kinase B (AKT) signaling, leading to reduced glucose uptake and compromised metabolic insulin actions [12]. Paradoxically, the mitogen-activated protein kinase (MAPK) pathway remains relatively preserved, promoting ovarian theca cell proliferation and steroidogenic activity [13]. This selective IR results in compensatory hyperinsulinemia, which augments ovarian androgen synthesis by stimulating cytochrome P450c17α and synergizing with luteinizing hormone (LH) [14]. Excess insulin also suppresses hepatic sex hormone-binding globulin (SHBG) synthesis; therefore, the bioavailability of circulating androgens increases [15].

The consequences of these molecular perturbations extend beyond ovarian steroidogenesis. Insulin directly influences follicular development, and chronic hyperinsulinemia disturbs folliculogenesis by accelerating early antral growth but impairing terminal maturation, resulting in follicular arrest and anovulation [16]. Additionally, systemic IR contributes to adipocyte hypertrophy, dyslipidemia, and altered adipokine secretion, reinforcing metabolic and inflammatory disturbances [17]. Emerging evidence indicates that IR-associated OS and mitochondrial dysfunction further propagate granulosa cell apoptosis and compromise oocyte competence [18]. Clinically, the presence of IR, beyond disruption of reproductive functions, highly elevates the risk of several metabolic syndromes, type 2 diabetes, and cardiovascular complications [19]. Therefore, IR represents both a diagnostic hallmark and therapeutic target in PCOS [11]. Interventions aimed at improving insulin sensitivity, including metformin and thiazolidinediones, have shown benefits but are often accompanied by adverse effects [20]. This has spurred interest in bioactive compounds, particularly flavonoids, which can modulate IRS-1/PI3K/AKT signaling, enhance insulin-responsive glucose transporter type 4 (GLUT4) translocation, and restore insulin responsiveness, offering a safer, multi-targeted therapeutic avenue [21].

### 2.2. Hyperandrogenism and Ovarian Dysfunction

Hyperandrogenism is a cardinal feature of PCOS, manifesting clinically as hirsutism, acne, and alopecia, and biochemically as elevated serum testosterone and androstenedione [22]. The ovarian theca cells are the primary source of androgen excess, driven by the intrinsic enzymatic hyperactivity of steroidogenic enzymes, such as cytochrome P450 family 17 subfamily A member 1 (CYP17A1), and augmented by hyperinsulinemia and elevated LH levels [23]. Insulin and LH act synergistically to enhance androgen biosynthesis, while the reduction of SHBG by hyperinsulinemia amplifies circulating free androgens [14].

At the ovarian level, androgen excess disrupts folliculogenesis, promoting early follicular recruitment but impeding selection of a dominant follicle, resulting in follicular arrest and anovulatory cycles [24]. Granulosa cell function is compromised through androgen-induced alterations in FSH receptor signaling, leading to impaired aromatase activity and reduced estradiol synthesis [25]. The cumulative effect is anovulation, menstrual irregularities, and subfertility. Moreover, hyperandrogenism contributes to altered oocyte competence, abnormal endometrial receptivity, and increased miscarriage risk [26]. The interaction between hyperandrogenism and metabolic dysfunction is reciprocal. IR potentiates androgen production, while androgens exacerbate visceral adiposity and metabolic risk [27]. Adipokines such as leptin, resistin, and adiponectin further modulate this endocrine–metabolic crosstalk and create a vicious cycle of androgen excess and metabolic dysregulation [28]. Pro-inflammatory cytokines amplify the ovarian hyperandrogenic milieu by enhancing steroidogenic enzyme expression [29].

Current therapeutic approaches, including combined oral contraceptives and anti-androgens, provide symptomatic relief but may not address underlying metabolic-endocrine interactions [30]. In this context, flavonoids, particularly isoflavones and phytoestrogens such as genistein and daidzein, have demonstrated anti-androgenic effects by modulating the expression of steroidogenic enzymes and restoring granulosa cell aromatase activity. By simultaneously improving insulin sensitivity and reducing inflammatory cytokine signaling, flavonoids offer a multi-level intervention against the hyperandrogenic ovarian phenotype of PCOS [31].

### 2.3. Adipokine–Cytokine Crosstalk

In PCOS women, adipose tissues are not merely an energy reservoir, but are active endocrine organs, secreting adipokines and cytokines that orchestrate metabolic and reproductive dysfunctions [32]. Altered adipokine profiles in PCOS are mainly characterized by elevated leptin, resistin, and visfatin, alongside reduced adiponectin levels [33]. Hyperleptinemia promotes ovarian dysfunction by impairing oocyte maturation and steroidogenesis, while hypoadiponectinemia reduces insulin sensitivity, aggravating systemic metabolic imbalance [34]. Resistin and visfatin contribute to inflammation and IR, further strengthening the pathogenic loop [35].

This adipokine dysregulation is closely intertwined with pro-inflammatory cytokines, particularly tumor necrosis factor-α (TNF-α), interleukin-6 (IL-6), and interleukin-1β (IL-1β) [29]. Chronic low-grade inflammation, now recognized as a hallmark of PCOS, arises from hypertrophic adipocytes, infiltrating macrophages, and activated immune cells in adipose tissue [36]. These cytokines promote serine phosphorylation of IRS-1 and activate stress kinases, thus interfering heavily with insulin signaling, perpetuating IR [37]. Within the ovary, cytokines dysregulate granulosa cell function, enhance androgen production, and impair folliculogenesis, contributing to anovulatory infertility [38]. The adipokine–cytokine axis thus represents the endocrine–immune–metabolic interface of PCOS, linking obesity, IR, and reproductive disturbances. Importantly, these mediators also increase the risk of systemic complications such as cardiovascular disease, metabolic syndrome, and gestational diabetes in PCOS women [19].

Conventional therapies have limited capacity to restore adipokine balance or resolve chronic inflammation. Flavonoids have emerged as promising modulators of this axis. Compounds like quercetin, luteolin, and naringenin have been shown to upregulate adiponectin, downregulate leptin and resistin, and suppress nuclear factor kappa-light-chain-enhancer of activated B cells (NF-κB) activation, thereby reducing cytokine secretion [39]. By targeting both metabolic and inflammatory arms of the crosstalk, flavonoids may provide a holistic therapeutic approach to ameliorate PCOS pathophysiology.

### 2.4. Oxidative Stress (OS) Induced Mitochondrial Dysfunction

OS and mitochondrial dysfunction are increasingly recognized as pivotal contributors to PCOS pathology [6]. Elevated reactive oxygen species (ROS) production, together with compromised antioxidant defences, creates a redox imbalance that affects multiple tissues, including the ovary, adipose tissue, and endometrium [40]. In granulosa cells, excessive ROS triggers apoptosis, disrupts mitochondrial membrane potential, and impairs steroidogenic activity, ultimately compromising oocyte competence [18,40]. Similarly, endometrial OS alters angiogenesis and reduces receptivity, contributing to implantation failure and recurrent pregnancy loss [41]. Mechanistically, hyperinsulinemia and hyperandrogenism exacerbate mitochondrial dysfunction by enhancing ROS generation and downregulating antioxidant enzymes such as superoxide dismutase (SOD), glutathione peroxidase (GPx), and catalase [6]. Mitochondrial deoxyribonucleic acid (DNA) damage, reduced adenosine triphosphate (ATP) production, and impaired mitochondrial biogenesis, marked by downregulation of regulators such as peroxisome proliferator-activated receptor-gamma coactivator (PGC)-1α, nuclear respiratory factor-1 (NRF1), and mitochondrial transcription factor A (TFAM), further compromise cellular bioenergetics, thereby exacerbating follicular arrest and ovulatory dysfunction in PCOS [18]. In adipose tissue, ROS-mediated stress activates pro-inflammatory transcription factors such as NF-κB, enhancing cytokine release and aggravating systemic inflammation and IR [32].

Clinical studies have documented elevated oxidative markers, including malondialdehyde and advanced oxidation protein products, along with a reduced total antioxidant capacity in women with PCOS [42,43]. These redox alterations are strongly correlated with IR, obesity, and infertility outcomes, highlighting OS as both a mechanistic driver and a potential biomarker of disease severity [18]. Targeting OS has gained therapeutic interest. While antioxidant supplements (such as N-acetylcysteine, resveratrol, and coenzyme Q10) have shown modest benefits in improving IR, oxidative markers, and ovulatory function in women with PCOS, flavonoids offer superior potential due to their dual ability to scavenge ROS and upregulate endogenous antioxidant systems [44]. Flavonoids such as catechins, quercetin, and anthocyanidins have been shown to enhance mitochondrial function, restore antioxidant enzyme activity, and prevent DNA damage [45]. By stabilizing mitochondrial dynamics and reducing granulosa cell apoptosis, flavonoids may improve oocyte quality and reproductive outcomes, positioning them as key agents in mitigating OS-driven pathology in PCOS. However, the clinical translation of flavonoid-based antioxidant therapy remains constrained by the limited number of large-scale randomized controlled trials (RCTs), poor bioavailability, and variable tissue-specific responses, which may affect their consistency and therapeutic efficacy in PCOS management.

## 3. Flavonoids: Structural Diversity and Medicinal Chemistry Aspects

### 3.1. Classification of Flavonoids

Flavonoids constitute a diverse class of polyphenolic secondary metabolites widely distributed across fruits, vegetables, cereals, legumes, and beverages such as tea and wine [46]. Structurally, they share a common diphenylpropane (C6-C3-C6) backbone consisting of two aromatic rings (A and B) linked through a heterocyclic pyran ring (C) [47]. Based on the degree of oxidation and substitution patterns on the C ring, flavonoids are classified into several subclasses [48], each exhibiting distinct pharmacological properties relevant to the pathophysiology of PCOS.

Flavones (e.g., luteolin, apigenin) are characterized by a double bond between C2 and C3, and a ketone group at C4 [49]. They are potent anti-inflammatory agents, frequently downregulating NF-κB and cyclooxygenase-2 (COX-2) expression, thereby counteracting cytokine-driven ovarian dysfunction [50]. Flavonols (e.g., quercetin, kaempferol) possess an additional hydroxyl group at C3, conferring strong antioxidant activity through hydrogen donation and metal-chelating properties [51]. These compounds modulate insulin sensitivity by enhancing IRS-1 phosphorylation and AMPK signaling [52]. Flavanones (e.g., naringenin, hesperidin), abundant in citrus fruits, exhibit insulin-sensitizing effects and improve lipid metabolism, particularly by regulating adiponectin secretion [53].

Isoflavones (e.g., genistein, daidzein) differ structurally by having the B ring attached to C3 of the heterocyclic ring [49]. As phytoestrogens, they bind to estrogen receptors, exerting hormone-modulatory and anti-androgenic actions, which are especially relevant to hyperandrogenism in PCOS [54]. Anthocyanidins (e.g., cyanidin, delphinidin), responsible for the pigmentation of berries and grapes, display strong free radical scavenging activity and improve endothelial function, potentially mitigating cardiovascular risks associated with PCOS [55]. Finally, chalcones (e.g., phloretin, isoliquiritigenin), open-chain flavonoids with unique α,β-unsaturated carbonyl groups, act as intermediates in flavonoid biosynthesis and exhibit anti-inflammatory and anti-obesity effects [56]. The structural diversity of flavonoids underpins their pleiotropic biological actions. The multiplicity of subclasses, each targeting distinct molecular nodes, ranging from insulin signaling and androgen synthesis to inflammatory and OS pathways, positions flavonoids as uniquely suited for addressing the multifactorial pathophysiology of PCOS. Understanding this classification provides a framework for linking structural motifs to pharmacological relevance in reproductive and metabolic disorders.

### 3.2. Pharmacokinetics and Bioavailability

Despite compelling preclinical evidence that flavonoids modulate PCOS-related pathways, their clinical translation is often hindered by poor pharmacokinetics and limited bioavailability [57]. Flavonoids are typically ingested in glycosylated or conjugated forms, which undergo extensive metabolism in the gastrointestinal tract. Hydrolysis by intestinal β-glucosidases or gut microbiota, particularly *Bacteroides*, *Clostridium*, *Eubacterium*, and *Lactobacillus* species, yields aglycones, which are subsequently absorbed and metabolized through phase II conjugation reactions. Once absorbed, flavonoids are rapidly conjugated in enterocytes and hepatocytes through glucuronidation, sulfation, and methylation, leading to low systemic concentrations of active metabolites [58].

The plasma half-lives of most flavonoids are relatively short (1–4 h), and tissue distribution is often restricted, with only trace amounts reaching target organs such as ovaries or adipose tissue [59]. Furthermore, inter-individual variability in gut microbiota composition significantly influences flavonoid metabolism, adding complexity to therapeutic outcomes [60]. These pharmacokinetic limitations partly explain the inconsistent efficacy observed in clinical studies of flavonoid supplementation in women with PCOS. Various steps have been undertaken to mitigate these challenges. Nano-formulations (e.g., liposomes, polymeric nanoparticles, and solid lipid nanoparticles) enhance solubility, protect flavonoids from enzymatic degradation, and improve tissue penetration [61]. Conjugation approaches, including glycoside modification and phospholipid complexes, have demonstrated improved stability and intestinal absorption [62]. Co-administration with bioenhancers, such as piperine, further reduces first-pass metabolism. Novel delivery platforms, including hydrogels and targeted nanoparticles, aim to achieve localized ovarian delivery, minimizing systemic clearance [63]. Importantly, the biological activity of flavonoids may not solely depend on the parent compound but also on their metabolites, which can retain or even surpass the bioactivity of the aglycones [64]. Thus, comprehensive pharmacokinetic and pharmacodynamic studies are crucial for elucidating effective doses and formulations that facilitate clinical translation. Without addressing these bioavailability hurdles, the therapeutic promise of flavonoids in PCOS may remain unrealized.

### 3.3. Structure–Activity Relationship (SAR)

**Figure 1:**
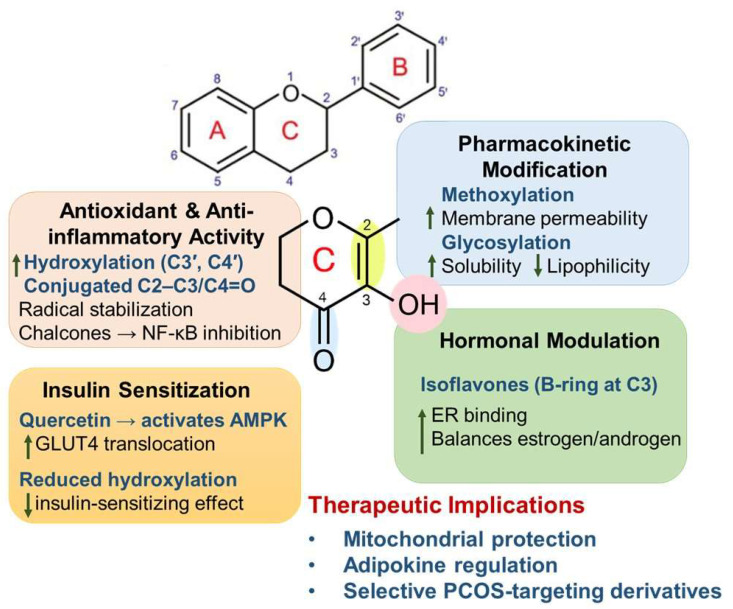
Structure–activity relationship (SAR) of flavonoids in polycystic ovary syndrome (PCOS). The diagram illustrates how specific structural features, such as hydroxylation, conjugated C2-C3/C4=O systems, and substituents like methoxy or glycosyl groups, govern key biological effects, including antioxidant and anti-inflammatory activity, insulin sensitization, and hormonal modulation. These structural modifications collectively influence pharmacokinetic behaviour and therapeutic potential in PCOS management.

SAR analyses provide critical insights into how specific structural motifs within flavonoids dictate their biological activity against PCOS-relevant pathways [65]. The presence and positioning of hydroxyl groups are critical in determining antioxidant and anti-inflammatory potency. For instance, hydroxylation at C3′ and C4′ on the B ring enhances radical scavenging capacity and metal-chelating activity, while a C2-C3 double bond conjugated with a C4 carbonyl (as in flavones and flavonols) increases electron delocalization, stabilizing reactive intermediates [66] (Figure 1).

The degree of hydroxylation also influences insulin-sensitizing effects. Quercetin, with multiple hydroxyl substitutions, strongly activates AMPK and enhances GLUT4 translocation in skeletal muscle, improving glucose uptake [67]. In contrast, flavonoids with fewer hydroxyl groups exhibit weaker effects [49]. Glycosylation generally reduces bioactivity by limiting lipophilicity and cell permeability, although it may enhance stability and solubility, underscoring the trade-off between activity and pharmacokinetics [68] (Figure 1).

Isoflavones, such as genistein, highlight the impact of structural rearrangements, with the B ring positioned at C3 rather than C2, which confers estrogen receptor affinity and phytoestrogenic activity [69]. This allows modulation of the androgen-estrogen balance, which is beneficial in PCOS-associated hyperandrogenism. Similarly, chalcones, lacking the C ring, possess an α,β-unsaturated carbonyl system that interacts with nucleophilic protein sites, thereby exerting potent anti-inflammatory activity via NF-κB suppression; these effects have been validated in multiple in vitro and rodent models, though evidence in PCOS models remains sparse [70]. Substitutions such as methoxylation can enhance metabolic stability and membrane permeability, but may reduce antioxidant potency [71]. On the other hand, glycosidic modifications (e.g., rutin) improve water solubility and bioavailability but attenuate direct free radical scavenging [72]. Emerging SAR studies emphasize the importance of conjugated systems and hydroxylation patterns in mitochondrial protection [73], granulosa cell survival, and adipokine regulation, mechanisms central to PCOS pathology. Integrating SAR knowledge with medicinal chemistry enables rational design of flavonoid derivatives or synthetic analogs with optimized activity and pharmacokinetic profiles. This approach offers the possibility of tailoring flavonoid-based therapeutics to selectively target IR, inflammatory cytokine expression, or androgen biosynthesis, thereby providing a precision strategy in PCOS management.

## 4. Flavonoids in Modulating Polycystic Ovary Syndrome (PCOS) Pathways

### 4.1. Effects on Insulin Resistance (IR) and Glucose Metabolism

As already discussed, IR is at the core of PCOS pathogenesis, and flavonoids demonstrate significant potential in improving glucose homeostasis through modulation of key signaling pathways [12]. Compounds such as quercetin, naringenin, luteolin, and catechins can significantly upregulate cellular metabolism by activating AMPK [74]. This activation leads to higher glucose uptake as AMPK facilitates GLUT4 translocation in skeletal muscle and adipose tissue, while simultaneously inhibiting hepatic gluconeogenesis [75]. This dual action contributes to improved systemic insulin sensitivity. Quercetin is particularly well-documented for enhancing phosphorylation of IRS-1 and restoring PI3K/AKT signaling, thereby overcoming one of the central defects in PCOS-related IR [76]. Naringenin produces comparable effects, while also offering additional benefits for lipid metabolism by regulating peroxisome proliferator-activated receptor gamma (PPARγ). This regulation enhances adipocyte differentiation and stimulates adiponectin secretion [77]. Similarly, luteolin and epigallocatechin-3-gallate (EGCG) have been shown to counteract oxidative stress–induced disruptions in insulin signaling by limiting ROS accumulation and strengthening antioxidant defences [78,79].

Beyond cellular mechanisms, flavonoids exert systemic effects by improving pancreatic β-cell function and reducing hyperinsulinemia, which in turn lessens the stimulatory effect of insulin on ovarian androgen production [80]. Animal studies in PCOS models treated with quercetin or EGCG demonstrate normalization of estrous cyclicity, improved insulin sensitivity, and reduced ovarian androgen synthesis [81,82]. Clinical trials, though limited in scale, have reported reduced fasting insulin and homeostatic model assessment for insulin resistance (HOMA-IR) indices following flavonoid supplementation in PCOS women [83]. Despite these promising outcomes, challenges to bioavailability limit clinical efficacy. Most flavonoids exhibit low plasma concentrations, and their rapid metabolism reduces sustained activity. Thus, advanced delivery systems such as nanoparticles and liposomal formulations are being investigated to optimize therapeutic outcomes. Collectively, flavonoids represent a promising class of nutraceuticals that target the metabolic underpinnings of PCOS, complementing conventional insulin-sensitizing agents like metformin while offering additional antioxidant and anti-inflammatory benefits.

### 4.2. Regulation of Adipokine Secretion

As mentioned before, adipokines serve as key mediators connecting metabolic disturbances to reproductive complications in PCOS [84]. Their dysregulated secretion, marked by increased levels of leptin, resistin, and visfatin alongside diminished adiponectin, further perpetuates chronic inflammation, insulin resistance, and ovarian dysfunction [33,85]. Flavonoids exert corrective influences on this imbalance of adipose tissue signaling pathways. Quercetin and luteolin have been shown to increase adiponectin secretion by activating AMPK and PPARγ, thereby improving insulin sensitivity and lipid metabolism [75]. Adiponectin upregulation also exerts anti-inflammatory effects by suppressing NF-κB activation, reducing systemic cytokine load [86]. In contrast, flavonoids like naringenin downregulate leptin expression in hypertrophic adipocytes, mitigating leptin resistance and restoring leptin sensitivity in hypothalamic and ovarian tissues. This is of particular relevance as hyperleptinemia is linked to impaired oocyte maturation and disrupted ovarian steroidogenesis [87].

Visfatin and resistin, pro-inflammatory adipokines implicated in IR and androgen excess [29], are downregulated by flavonoid intervention. Experimental evidence indicates that luteolin reduces visfatin-induced NF-κB activation, while catechins suppress resistin-mediated cytokine release [88]. By restoring a favourable adipokine milieu, flavonoids alleviate metabolic disturbances and reduce ovarian hyperandrogenism. Animal models of PCOS treated with quercetin or EGCG have demonstrated improved adipokine profiles, decreased ovarian cyst formation, and normalized estrous cycles [89,90]. Limited clinical trials report reductions in leptin and increases in adiponectin following flavonoid supplementation, supporting translational relevance [91]. However, variability in dosage, formulation, and study populations restricts definitive conclusions. Therefore, flavonoids appear to act as modulators of the adipokine–cytokine axis, bridging metabolic and reproductive improvements in PCOS. Their ability to restore adipokine balance provides a mechanistic basis for their therapeutic promise, particularly in obese PCOS phenotypes where adipose tissue dysfunction is a key driver of pathophysiology.

### 4.3. Cytokine Suppression and Anti-Inflammatory Effects

Chronic low-grade inflammation is often associated with PCOS, which contributes to higher titers of pro-inflammatory cytokines such as IL-6, TNF-α, and IL-1β [36]. These cytokines impair insulin signaling, enhance androgen biosynthesis, and disrupt ovarian folliculogenesis [92]. Flavonoids exert strong anti-inflammatory effects by targeting key molecular pathways that regulate cytokine production and signaling [66]. A central mechanism is the inhibition of NF-κB activation, a transcription factor that regulates the expression of multiple inflammatory mediators [93]. Quercetin, luteolin, and EGCG suppress NF-κB nuclear translocation, thereby downregulating TNF-α, IL-6, and COX-2 expression [94,95,96]. Isoflavones such as genistein modulate inflammatory responses by inhibiting MAPK signaling cascades, further reducing cytokine release from immune and adipose cells [97]. Flavonoids also regulate inflammasome activation. Studies have shown that luteolin and apigenin inhibit NLRP3 (nucleotide-binding domain, leucine-rich–containing family, pyrin domain–containing-3) inflammasome assembly, reducing IL-1β production, a cytokine strongly implicated in ovarian dysfunction [98]. Additionally, flavonoids restore the balance between pro- and anti-inflammatory cytokines by upregulating interleukin-10 (IL-10), an anti-inflammatory mediator that promotes ovarian immune homeostasis [99].

Preclinical evidence supports these effects: in rodent PCOS models, flavonoid supplementation reduces ovarian infiltration of immune cells, normalizes follicular morphology, and restores ovulatory cycles [57]. Clinical studies, though limited, report significant reductions in serum TNF-α and C-reactive protein (CRP) levels after quercetin or isoflavone supplementation in women with PCOS [100]. The anti-inflammatory properties of flavonoids extend beyond cytokine suppression, encompassing inhibition of adhesion molecules and chemokines that perpetuate immune cell recruitment [66]. These actions collectively restore ovarian immune balance, improve follicular growth, and enhance reproductive outcomes. Given that inflammation underpins both metabolic and reproductive dysfunctions in PCOS, flavonoids offer a comprehensive therapeutic modality by targeting cytokine-driven pathways that conventional treatments often neglect.

### 4.4. Antioxidant and Mitochondrial Protective Roles

OS is intricately linked to PCOS, with elevated ROS and impaired antioxidant defences compromising granulosa cell function, oocyte competence, and endometrial receptivity [6]. Flavonoids also exhibit potent antioxidant properties, with both direct and indirect mechanisms of action, making them attractive candidates for mitigating redox imbalances in PCOS [78]. Direct ROS scavenging is conferred by hydroxyl groups on flavonoid structures, which donate hydrogen atoms to neutralize free radicals [73]. Flavonols, such as quercetin and kaempferol, possess strong radical-quenching activity, which prevents lipid peroxidation and DNA damage in ovarian cells [101,102]. Indirectly, flavonoids upregulate endogenous antioxidant enzymes, including SOD, catalase, and GPx, through activation of the nuclear factor erythroid 2-related factor 2 (Nrf2)-antioxidant responsive element (ARE) pathway, thereby strengthening cellular resilience against oxidative insults [103]. Mitochondrial protection is another crucial facet of flavonoid action [79]. Quercetin and EGCG restore mitochondrial membrane potential, enhance ATP production, and reduce mitochondrial DNA damage in granulosa cells and oocytes [81]. Luteolin has been shown to modulate mitochondrial biogenesis by upregulating PGC-1α, thereby improving bioenergetic efficiency. By stabilizing mitochondrial function, flavonoids reduce granulosa cell apoptosis and improve oocyte maturation, a critical determinant of reproductive success in PCOS [79,104].

Animal studies have corroborated these findings, demonstrating reduced ovarian ROS levels, improved follicular development, and enhanced fertility outcomes following flavonoid administration [57]. Clinical trials are still limited, but suggest improved OS markers and menstrual regularity in PCOS women supplemented with flavonoids [105]. Importantly, flavonoids also counteract OS in extra-ovarian tissues, reducing vascular dysfunction and systemic inflammation, which are prevalent comorbidities in PCOS [106]. Their dual role as antioxidants and mitochondrial protectants highlights their potential to address both reproductive and metabolic dysfunctions simultaneously, reinforcing their position as multi-target agents in PCOS therapy.

### 4.5. Modulation of Hyperandrogenism and Hormonal Imbalance in PCOS

Hyperandrogenism underlies many of the reproductive manifestations of PCOS, including anovulation, follicular arrest, and menstrual irregularities [107]. Flavonoids, particularly isoflavones and phytoestrogens, demonstrate hormone-modulating properties that directly attenuate androgen excess [108]. Isoflavones such as genistein and daidzein, structurally similar to estrogens, bind to estrogen receptors (ER-α and ER-β), exerting selective estrogen receptor modulator (SERM)-like effects. By enhancing estrogenic signaling, they counterbalance androgen-driven suppression of granulosa cell aromatase, thereby restoring estradiol synthesis and follicular maturation. Genistein also inhibits ovarian CYP17A1 activity, reducing androgen biosynthesis in theca cells [109,110].

Beyond isoflavones, quercetin and apigenin reduce androgen receptor (AR) expression and downregulate steroidogenic enzymes, thereby diminishing androgen action at the ovarian level [31]. Naringenin has demonstrated the ability to increase SHBG levels, reducing bioavailable testosterone and alleviating hyperandrogenic symptoms [31]. Additionally, flavonoids modulate hypothalamic–pituitary signaling, normalizing LH/FSH ratios that are frequently altered in PCOS, thereby improving ovulatory function [10]. Preclinical studies in letrozole- and dehydroepiandrosterone (DHEA)-induced PCOS models reveal that flavonoid supplementation reduces serum testosterone, restores estrous cyclicity, and improves ovarian morphology [81]. Clinical evidence, though modest, indicates that isoflavone supplementation reduces total and free testosterone while improving menstrual regularity in PCOS women [111]. Flavonoids also enhance endometrial receptivity by modulating estrogen-progesterone balance, potentially improving implantation success in PCOS-related infertility [112]. The combined anti-androgenic and hormone-regulatory effects highlight their ability to target both the biochemical and clinical features of PCOS [81]. Importantly, unlike conventional anti-androgens, flavonoids provide these benefits with a favourable safety profile, supporting their role as nutraceutical adjuncts in managing hyperandrogenic manifestations of PCOS. Clinical relevance of flavonoids in PCOS, including their physiological targets and therapeutic outcomes, is presented in Table 1.

## 5. Translational and Clinical Evidence

### 5.1. Preclinical Evidence in Animal Models of PCOS

Animal models have been pivotal in elucidating the mechanistic roles of flavonoids in PCOS pathophysiology [89,108]. Rodent models induced by letrozole, DHEA, or IR mimetics closely replicate features of human PCOS, including hyperandrogenism, anovulation, and metabolic dysfunction [108,115]. These models provide a robust platform for assessing the therapeutic efficacy of flavonoids. Quercetin has been extensively studied in letrozole-induced PCOS rats, where it reduces ovarian cyst formation, restores estrous cyclicity, and lowers serum testosterone levels [54,108]. Mechanistically, quercetin upregulates AMPK signaling, improves insulin sensitivity, and downregulates ovarian CYP17A1 expression, leading to reduced androgen biosynthesis [31]. Similarly, naringenin has shown significant effects in DHEA-induced models, improving glucose tolerance, increasing adiponectin levels, and reducing systemic inflammation through suppression of NF-κB signaling [77,87]. Catechins, particularly EGCG, have demonstrated dual benefits by attenuating OS and improving mitochondrial function in ovarian tissue [78,94]. EGCG supplementation normalizes follicular development and enhances oocyte maturation, underscoring its reproductive benefits [94]. Isoflavones such as genistein have exhibited estrogen receptor-mediated effects, restoring hormonal balance and reducing anovulation in rodent PCOS models [108].

Beyond reproductive improvements, preclinical studies have highlighted the systemic metabolic effects of flavonoids, including reduced dyslipidemia, improved hepatic steatosis, and modulation of adipokine profiles. Importantly, flavonoid supplementation is associated with reductions in inflammatory cytokines such as TNF-α and IL-6, aligning with their role in targeting chronic low-grade inflammation characteristic of PCOS [66]. Therefore, preclinical data consistently demonstrate the capacity of flavonoids to modulate multiple pathogenic pathways, ranging from IR and OS to hyperandrogenism and inflammation. However, heterogeneity in animal models, dosing regimens, and bioavailability remains a limitation. Standardization of experimental protocols and translational studies bridging rodent and human physiology is essential for clinical application.

### 5.2. Human Clinical Studies and Trials

Clinical research on flavonoids in PCOS is growing, although it is limited by small sample sizes, short intervention durations, and variable formulations. Among flavonoids, quercetin, resveratrol, and isoflavones have been most frequently evaluated in RCTs. Quercetin supplementation (500–1000 mg/day for 8–12 weeks) has been shown to significantly improve insulin sensitivity, lower fasting glucose, and reduce serum testosterone in women with PCOS [113]. These effects are attributed to enhanced IRS-1/PI3K/AKT signaling and downregulation of CYP17A1. Some trials also reported improvements in menstrual regularity and ovulatory frequency, highlighting reproductive benefits [116]. Resveratrol, although not a classical flavonoid, is structurally related and has demonstrated substantial promise [41,73]. Clinical studies reveal reductions in serum testosterone, improved insulin sensitivity, and modulation of inflammatory cytokines. Notably, resveratrol has been associated with reduced ovarian theca cell androgen production, offering a direct anti-androgenic mechanism [117]. Isoflavones, particularly genistein and daidzein, have been primarily investigated in Asian populations, where higher habitual soy intake and phytoestrogen exposure may partly explain the observed reductions in androgen levels and improvements in lipid profiles; however, such ethnic and dietary variability complicates interpretation and may limit the generalizability of these findings to other populations [118].

Despite these encouraging outcomes, several limitations exist. Heterogeneity in PCOS phenotypes, ethnicity, and BMI status influences treatment responses. Variations in flavonoid formulation (aglycone vs. glycoside, capsule vs. dietary source) and bioavailability further complicate the reproducibility of results. Moreover, most studies have short follow-up durations, which are insufficient to assess the long-term effects on fertility or cardiometabolic outcomes. Meta-analyses suggest that flavonoid supplementation exerts moderate but clinically relevant improvements in metabolic and hormonal parameters [83]. However, the evidence remains insufficient for guideline recommendations. Large-scale, well-designed RCTs with standardized formulations, diverse populations, and longer durations are essential to establish flavonoids as evidence-based adjuncts in PCOS management.

### 5.3. Safety and Toxicological Considerations

While flavonoids are generally regarded as safe due to their natural dietary origin, safety and toxicological considerations are critical for their translation into PCOS therapeutics. The majority of clinical studies report good tolerability, with mild gastrointestinal disturbances being the most common adverse effect. However, dose, formulation, and duration significantly influence safety outcomes. High-dose supplementation of certain flavonoids, such as quercetin and EGCG, has been associated with hepatotoxicity in rare cases, particularly when consumed in concentrated extracts rather than dietary forms [119]. Isoflavones, due to their phytoestrogenic activity, raise concerns regarding endocrine modulation, particularly in women with hormone-sensitive conditions [120]. Long-term safety data in reproductive-age women remain sparse, warranting caution in extrapolating findings from general populations.

Another key consideration is reproductive toxicity. While animal models generally report improvements in fertility parameters, high doses of some flavonoids have shown potential interference with steroidogenesis or fetal development, though these effects are dose-dependent and not consistently reproduced [121]. Interactions with conventional PCOS therapies, such as metformin, clomiphene, or oral contraceptives, are underexplored and could influence drug metabolism or efficacy. Bioavailability-enhancing formulations, including nanoparticles and conjugates, may alter pharmacokinetics and toxicity profiles. Therefore, rigorous toxicological assessments are necessary for novel delivery systems prior to clinical translation. Additionally, inter-individual variability in gut microbiota and genetic polymorphisms in metabolizing enzymes can influence safety and efficacy, underscoring the importance of personalized approaches [60]. Therefore, flavonoids exhibit an excellent safety profile at dietary or moderate supplemental doses, with potential risks emerging at pharmacological levels or with prolonged use. Careful dose optimization, long-term monitoring, and systematic safety evaluations are crucial for their incorporation into clinical practice for PCOS. Establishing standardized safety thresholds will enhance confidence in their therapeutic deployment. Translational and clinical evidence of flavonoids in PCOS management, as evidenced from animal models and human trials, is presented in Table 2.

## 6. Integrative Therapeutic Perspective

### 6.1. Flavonoids as Adjunct to Standard Therapies

Current therapeutic regimens for PCOS, including metformin, clomiphene citrate, and letrozole, primarily target IR, ovulation induction, and hormonal regulation [20,122]. However, these treatments often demonstrate variable efficacy, are associated with adverse effects such as gastrointestinal discomfort (metformin) or multiple gestations (clomiphene), and do not adequately address the chronic inflammatory and OS components of PCOS [123,124]. This has prompted growing interest in flavonoids as potential adjuncts to conventional therapies, offering a multi-targeted approach to disease management [105]. Preclinical studies reveal that flavonoids enhance the therapeutic efficacy of metformin by synergistically activating AMPK and improving GLUT4-mediated glucose uptake [75]. Quercetin, for example, complements metformin in reducing hyperinsulinemia and ovarian androgen production [114]. Similarly, isoflavones exhibit estrogen receptor-modulating properties that may augment clomiphene or letrozole by improving endometrial receptivity and increasing ovulatory rates [125]. Moreover, catechins and naringenin have demonstrated lipid-lowering effects that complement the metabolic benefits of metformin and lifestyle interventions [77,78].

Beyond metabolic synergy, flavonoids confer unique benefits by mitigating systemic inflammation and OS [66,103]. This dual role can reduce the risk of cardiovascular and reproductive comorbidities, which are insufficiently addressed by standard pharmacotherapies [118]. Clinical trials suggest that flavonoid supplementation enhances the overall response to conventional drugs, improving menstrual regularity, reducing androgen levels, and ameliorating IR more effectively than monotherapy [105]. Nevertheless, integration into clinical practice requires caution. Potential pharmacokinetic interactions, such as flavonoid-mediated modulation of cytochrome P450 enzymes, may alter drug metabolism [126]. Standardization of flavonoid formulations, dosing, and duration is also necessary to avoid variability in outcomes. Despite these challenges, the adjunctive use of flavonoids holds strong promise for optimizing PCOS therapy, minimizing drug doses, reducing side effects, and improving patient adherence. Although preliminary evidence suggests potential synergy between flavonoids and standard therapies, comprehensive clinical trials directly evaluating these combinations are still limited, representing an important area for future research.

### 6.2. Nutraceutical and Functional Food Applications

The recognition of diet as a modifiable factor in PCOS pathophysiology has positioned flavonoids as prime candidates for nutraceutical and functional food applications [127]. Unlike synthetic drugs, dietary flavonoids can be incorporated into everyday nutrition, providing a safe, non-pharmacological approach to managing both metabolic and reproductive manifestations of PCOS. Flavonoids are widely available in foods such as berries (anthocyanidins), soy products (isoflavones), citrus fruits (naringenin, hesperidin), onions and apples (quercetin), and green tea (catechins) [128]. Epidemiological evidence suggests that diets rich in flavonoid-containing foods are associated with reduced risk of metabolic syndrome, improved insulin sensitivity, and better cardiovascular health [129], outcomes directly relevant to PCOS. Nutraceutical formulations, including concentrated extracts or standardized supplements, further enhance flavonoid intake beyond dietary levels, providing targeted therapeutic benefits [130]. Functional foods fortified with flavonoids are emerging as innovative strategies. For instance, soy-based products enriched with isoflavones have demonstrated improvements in menstrual cyclicity and androgen levels in women with PCOS [54,111]. Similarly, green tea extracts incorporated into beverages or supplements have been linked to reduced BMI, improved lipid metabolism, and enhanced ovulatory function [131]. The ability of flavonoids to simultaneously influence multiple biological pathways, insulin signaling, adipokine secretion, OS, and androgen synthesis, makes them ideal candidates for dietary-based interventions [65].

Despite this promise, challenges persist. The variability in flavonoid content among foods, influenced by cultivation, processing, and storage, complicates the consistency of intake. Additionally, individual differences in metabolism, largely shaped by gut microbiota, lead to heterogeneous responses. Nutraceutical interventions must therefore be evidence-based, with standardized formulations and dosing regimens supported by clinical trials. Therefore, incorporating flavonoids into nutraceutical and functional food strategies aligns with the growing emphasis on lifestyle-based management of PCOS. By offering safe, sustainable, and patient-friendly interventions, flavonoids can complement pharmacological approaches and empower women with PCOS to actively manage their condition through dietary choices.

### 6.3. Novel Formulations and Delivery Systems

One of the principal barriers limiting the therapeutic utility of flavonoids in PCOS is their poor bioavailability [10]. Rapid metabolism, low solubility, and limited tissue distribution restrict their systemic activity, resulting in inconsistent clinical outcomes [132]. To overcome these challenges, novel formulations and delivery systems are being developed to optimize pharmacokinetics, enhance tissue targeting, and prolong bioactivity. Nanoparticle-based delivery systems, including polymeric nanoparticles, solid lipid nanoparticles, and nanostructured lipid carriers, have demonstrated significant potential in improving flavonoid solubility, stability, and absorption [133]. For instance, quercetin-loaded nanoparticles exhibit enhanced bioavailability and greater insulin-sensitizing effects compared to free quercetin in experimental PCOS models [134]. Liposomal formulations (such as quercetin-loaded liposomes) provide another strategy by encapsulating flavonoids within phospholipid bilayers, protecting them from enzymatic degradation and facilitating targeted delivery to ovarian and adipose tissues [135]. Micelle-based systems and phytosome complexes have been developed to improve the oral absorption of hydrophobic flavonoids [136]. EGCG-phytosomes, for example, achieve higher plasma concentrations and prolonged half-life, enabling sustained antioxidant and anti-inflammatory effects [137]. Hydrogels and controlled-release formulations further allow steady flavonoid release, minimizing fluctuations in plasma levels and maximizing therapeutic efficacy [138]. Targeted delivery approaches are also under investigation. Surface-modified nanoparticles conjugated with ovarian-specific ligands may enable localized delivery of flavonoids, reduce systemic clearance, and enhance direct effects on ovarian function. Such strategies could optimize therapeutic benefits while minimizing risks of systemic toxicity. While preclinical evidence supports the efficacy of advanced formulations, translation into clinical practice remains limited. While nano-formulations have significantly improved the solubility and bioavailability of flavonoids, targeted ovarian delivery remains largely theoretical, with limited experimental validation. Moreover, the potential for nanoparticle-related toxicity or altered pharmacodynamics warrants careful safety assessment in future studies. Regulatory challenges, safety concerns related to novel careers, and the high cost of development hinder widespread adoption. Nonetheless, the development of innovative delivery platforms represents a critical step toward realizing the full therapeutic potential of flavonoids in PCOS management. By overcoming bioavailability hurdles, these novel systems may transform flavonoids from promising nutraceuticals into clinically viable therapeutic agents.

**Figure 2:**
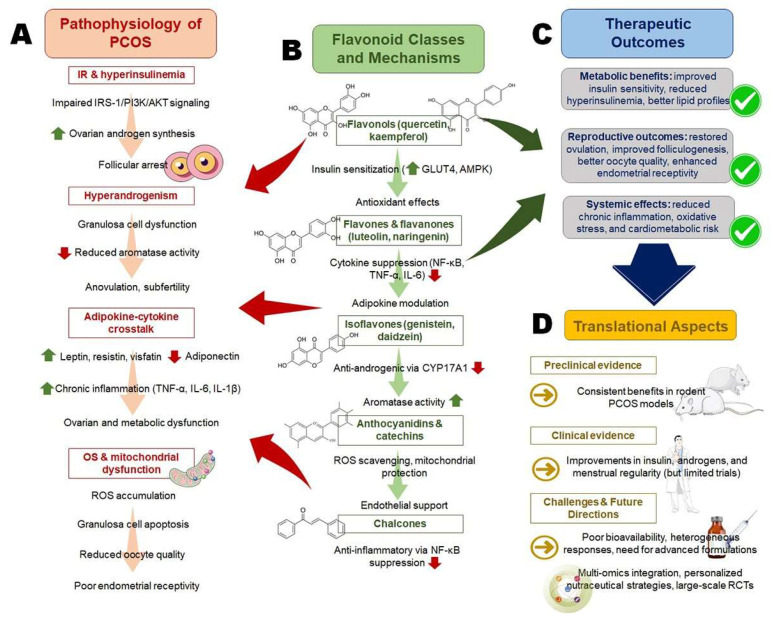
Multi-level interplay between polycystic ovarian syndrome (PCOS) pathophysiology, flavonoid mechanisms, and therapeutic outcomes. (**A**) IR and hyperinsulinemia disrupt IRS-1/PI3K/AKT signaling, elevate androgens, and impair folliculogenesis, while dysregulated adipokines and oxidative stress promote inflammation and poor oocyte quality. (**B**) Flavonols enhance insulin sensitivity and antioxidant defense; flavones/flavanones suppress cytokines and modulate adipokines; isoflavones reduce androgens and support aromatase; anthocyanidins and catechins protect mitochondria; chalcones inhibit NF-κB. (**C**) Outcomes include improved metabolism, hormonal balance, and fertility. (**D**) Translational data support preclinical efficacy and emerging clinical benefits, though bioavailability and long-term validation remain challenges.

Figure 2 presents the multi-level interplay between PCOS pathophysiology, flavonoid mechanisms, therapeutic outcomes, and translational perspectives.

## 7. Future Directions and Research Gaps

Despite compelling preclinical and emerging clinical evidence, the therapeutic application of flavonoids in PCOS remains in its early stages, hindered by several unresolved challenges. Future research must address these gaps through multidisciplinary and translational approaches to establish flavonoids as credible interventions in the management of PCOS. A critical priority is the execution of large-scale RCTs with standardized flavonoid formulations, dosages, and intervention durations. Most existing clinical studies are limited by small cohorts, heterogeneous PCOS phenotypes, and short follow-up periods, which restrict their generalizability. Robust RCTs, stratified by phenotype, ethnicity, and metabolic status, are essential to validate efficacy and safety across diverse populations. Equally important is the adoption of personalized medicine approaches. PCOS is a heterogeneous syndrome with variable contributions from IR, hyperandrogenism, inflammation, and OS. Genetic polymorphisms in flavonoid-metabolizing enzymes (e.g., uridine-5ʹ-diphosphate-glucuronosyltransferases or UGTs, catechol-O-methyltransferase or COMT) and inter-individual variability in gut microbiota composition significantly influence flavonoid bioactivity and metabolism. Incorporating pharmacogenomic and microbiome profiling could guide individualized supplementation strategies, optimizing therapeutic outcomes.

Advances in multi-omics technologies (metabolomics, transcriptomics, proteomics, and epigenomics) provide unprecedented opportunities to dissect the molecular mechanisms of flavonoid action in PCOS [139]. Systems biology approaches can reveal how flavonoids modulate crosstalk among metabolic, endocrine, and immune pathways, while identifying novel biomarkers of response. Integration of multi-omics datasets with machine learning may enable predictive modeling of patient-specific benefits. Further research is needed to develop novel formulations and delivery systems that overcome bioavailability barriers. Nanoparticles, liposomes, and phytosomes must undergo rigorous pharmacokinetic and toxicological evaluations to ensure clinical applicability. Additionally, long-term safety studies are lacking; the potential reproductive and endocrine effects of chronic flavonoid use must be carefully examined, particularly in women of reproductive age. Finally, incorporation of flavonoids into clinical guidelines and nutraceutical policies demands strong evidence of efficacy, safety, and cost-effectiveness. Collaborative efforts between clinicians, pharmacologists, nutritionists, and regulatory agencies will be vital. Therefore, the future of flavonoids in PCOS therapy lies in moving from observational promise to evidence-based integration, leveraging personalized strategies, multi-omics insights, and advanced formulations to maximize translational impact.

## 8. Conclusions

PCOS exemplifies a complex disorder where metabolic, endocrine, and inflammatory axes converge to drive reproductive dysfunction and long-term cardiometabolic risks. Conventional therapies, while beneficial in symptom management, are limited by partial efficacy, side effects, and inability to address the multifactorial nature of the syndrome. Flavonoids, owing to their structural diversity and pleiotropic actions, emerge as promising multi-target agents capable of modulating IR, adipokine–cytokine imbalance, OS, and hyperandrogenism. Preclinical evidence consistently demonstrates their capacity to restore ovarian morphology, improve ovulation, and normalize metabolic markers. Early clinical studies suggest that flavonoids can positively modulate insulin sensitivity, hormonal profiles, and inflammatory parameters. However, translation into routine clinical practice requires overcoming challenges related to bioavailability, inter-individual variability, and limited long-term safety data. Integration of advanced delivery systems, multi-omics analyses, and personalized approaches will be pivotal in refining their therapeutic utility. Ultimately, flavonoids hold significant potential as adjuncts to pharmacological therapies and as nutraceutical strategies, offering a holistic approach to PCOS management that aligns with both metabolic and reproductive health outcomes.

## Figures and Tables

**Table 1:** Clinical relevance of flavonoid subclasses in polycystic ovary syndrome (PCOS): pathophysiological targets and therapeutic outcomes.

Flavonoid Subclass (Examples)	Primary Molecular Targets	Pathophysiological Pathway in PCOS	Reported Clinical/Experimental Outcomes	References
Flavonols (Quercetin, Kaempferol)	IRS-1/PI3K/AKT, AMPK	Insulin resistance, hyperinsulinemia	↓ Fasting insulin, improved HOMA-IR, ↓ serum testosterone, restored ovulation	[76,81,113,114]
Flavones (Luteolin, Apigenin)	NF-κB, COX-2, NLRP3 inflammasome	Chronic inflammation, cytokine excess	↓ TNF-α, IL-6, IL-1β; restored ovarian folliculogenesis	[50,95,98,100]
Flavanones (Naringenin, Hesperidin)	PPARγ, adipokine regulation	Adipose tissue dysfunction, dyslipidemia	↑ Adiponectin, ↓ leptin; improved lipid profiles, improved ovulation in models	[53,77,87]
Isoflavones (Genistein, Daidzein)	Estrogen receptors, CYP17A1	Hyperandrogenism, follicular arrest	↓ Serum androgens, ↑ estradiol, improved menstrual cyclicity, improved endometrial receptivity	[54,109,111]
Catechins/Anthocyanidins (EGCG, Cyanidin)	ROS scavenging, Nrf2 pathway, mitochondrial biogenesis	Oxidative stress, mitochondrial dysfunction	↓ ROS, ↑ antioxidant enzymes, improved oocyte quality, reduced cystic follicles	[78,79,94,104]

**Table 2:** Translational landscape of flavonoids in polycystic ovary syndrome (PCOS): Evidence from animal models and human trials.

Flavonoid	Model/Population Studied	Dose and Duration	Major Findings	Clinical Implications	References
Quercetin	Letrozole/DHEA rat models; women with PCOS	50–150 mg/kg in rodents; 500–1000 mg/day in women for 8–12 weeks	↓ Serum testosterone, improved insulin sensitivity, restored ovulation	Potential adjunct to metformin for metabolic + reproductive benefits	[81,89,113,114]
Naringenin	DHEA-induced PCOS rats	50–100 mg/kg for 4–6 weeks	↑ Adiponectin, ↓ systemic inflammation, improved estrous cycles	Targeting obese/insulin-resistant PCOS phenotypes	[77,87,90]
EGCG (green tea catechin)	PCOS rodent models; small RCTs	50–100 mg/kg in rodents; 300–600 mg/day in humans	↓ ROS, improved folliculogenesis, reduced BMI, improved ovulation	Nutraceutical for oxidative-stress-driven PCOS	[78,82,94,105]
Isoflavones (Genistein/Daidzein)	Rodent PCOS models; Asian PCOS women	30–50 mg/day for 12 weeks	↓ Total testosterone, ↑ estradiol, improved menstrual regularity	Dietary intervention with hormone-modulatory benefits	[54,108,111,118]
Resveratrol (flavonoid-like stilbene)	PCOS women; granulosa cell studies	1000–1500 mg/day for 3 months	↓ Theca cell androgen production, improved insulin sensitivity, ↓ inflammatory cytokines	Potential anti-androgenic nutraceutical, but bioavailability limits use	[41,73,117]
Luteolin	Rodent PCOS models; in vitro ovarian/adipose cells	25–50 mg/kg (rodents), 4–6 weeks (typical preclinical)	↓TNF-α, IL-6, IL-1β via NF-κB/NLRP3 inhibition; ↑AMPK activity; improved insulin signaling; reduced ovarian inflammatory infiltration; improved folliculogenesis	Anti-inflammatory + insulin-sensitizing candidate; supports ovulatory function and metabolic control in PCOS	[79,95,104]
Apigenin	Rodent PCOS models; granulosa/adipocyte cultures	25–50 mg/kg (rodents), 4–8 weeks	Suppresses NF-κB/COX-2; mitigates oxidative stress; improves HOMA-IR surrogates in models; supports granulosa cell survival	Anti-inflammatory/antioxidant with potential to improve IR and ovarian microenvironment	[31,98]

## Data Availability

No new data were created.

## Abbreviations

AKT	Protein kinase B
AR	Androgen receptor
ARE	Antioxidant-responsive element
ATP	Adenosine triphosphate
COMT	Catechol-O-methyltransferase
COX-2	Cyclooxygenase-2
CRP	C-reactive protein
CYP17A1	Cytochrome P450 family 17 subfamily A member 1
DHEA	Dehydroepiandrosterone
DNA	Deoxyribonucleic acid
EGCG	Epigallocatechin-3-gallate
ER	Estrogen receptor
GPx	Glutathione peroxidase
HOMA-IR	Homeostatic model assessment for insulin resistance
IL-1β	Interleukin-1β
IL-6	Interleukin-6
IL-10	Interleukin-10
IR	Insulin resistance
IRS-1	Insulin receptor substrate-1
LH	Luteinizing hormone
MAPK	Mitogen-activated protein kinase
NF-κB	Nuclear factor kappa-light-chain-enhancer of activated B cells
NLRP3	Nucleotide-binding domain, leucine-rich-containing family, pyrin domain-containing-3
Nrf2	Nuclear factor erythroid 2-related factor 2
OS	Oxidative stress
PCOS	Polycystic ovary syndrome
PI3K	Phosphatidylinositol 3-kinase
PPARγ	Peroxisome proliferator-activated receptor γ
RCT	Randomized controlled trial
ROS	Reactive oxygen species
SAR	Structure–activity relationship
SERM	Selective estrogen receptor modulator
SHBG	Sex hormone-binding globulin
SOD	Superoxide dismutase
TFAM	Mitochondrial transcription factor A
TNF-α	Tumor necrosis factor-α
UGT	Uridine-5ʹ-diphosphate-glucuronosyltransferases
